# EXPERIENCE OF UNFAIR TREATMENT IN HEALTHCARE SETTING AND RELATED STRESS AMONG OLDER AMERICANS: RACIAL/ETHNIC GAPS

**DOI:** 10.1093/geroni/igac059.1834

**Published:** 2022-12-20

**Authors:** Eun-Hye Yi, Michin Hong, Cherish Bolton

**Affiliations:** Indiana State University, Terre Haute, Indiana, United States; Indiana University, Indianapolis, Indiana, United States; Indiana State University, Terre Haute, Indiana, United States

## Abstract

Guided by the intersectionality framework, this study examined the experience of unfair treatment in healthcare settings over lifetime and related stress. A subsample drawn from the California Health and Interview Survey 2017, including residents age 55 or higher, was used (N=12,261). Significant differences existed in unfair treatment and corresponding stress among racial/ethnic groups, including Whites, African Americans (AA), Hispanics, and Asian Americans (AS). Using weighted chi-square tests, we found that most Whites (75.99%) never experienced unfair treatment, while around 60% of AA answered never. Whites tended to feel extreme stress more when mistreated (23.47%) than Hispanics (14.83%) and AS (15.69%). Weighted logistic regression analyses revealed that younger older adults with lower mental health were more likely to experience unfair treatment across all race/ethnic groups. Intersectional factors contributing to unfair treatment experience were identified for each race/ethnic group. Being a female, living in poverty, poor health, being a naturalized citizen, and living in an urban area were factors for Whites while having higher education was a factor for AA. Mental health was associated with extreme stress for the unfair treatment in all racial/ethnic groups. Different contributors to the stress were found by race/ethnicity. Gender, poverty, citizenship, and length of staying in the U.S. were significant for Whites. For AA, poverty, healthcare insurance, and obesity were significant, and for AS, physical health and obesity were. This study highlights the importance of culturally/ethnically sensitive approaches shaping interventions and policies to enhance awareness about unfair treatment and preventing discrimination toward diverse older adults.

